# Supplementary material for: Using Machine Learning to Develop Smart Reflex Testing Protocols
Source: ArXiv. 2023 Feb 1:arXiv:2302.00794v1. Preprint. [Version 1] (PMC9915755)
Supplement: Supplement 1 [file NIHPP2302.00794v1-supplement-1.pdf]

**Supplement A: Association between ferritin results and predicted probabilities**

*Methods:* For cases where ferritin testing was performed concurrent with or within 30 days after the CBC, we assessed the ferritin test results to look for an association between predicted probability of ferritin ordering and results. We grouped cases by decile based on their test partition predicted probability of getting a ferritin. For cases that were in the test partition on multiple runs, the median predicted probability was used. For each decile, we calculated the proportion of ferritin results that were abnormally low (below our laboratory's reference cutoffs of 10mg/dL for females and 30mg/dL for males). This portion of the analysis was performed in R [9] with confidence intervals calculated using the Wilson method for binomial confidence intervals as implemented in the R binom package. Results were plotted in R using the ggplot2 package [10].

*Results:*

As shown (Figure S1), patients in whom the model predicted a particularly low likelihood of having a ferritin ordered (e.g., decile 1 in Figure 4) very rarely had abnormally low ferritin results. Presumably in most ferritin orders in patients with predicted likelihoods in the first decile were unnecessary and would have been avoided using an optimized reflex testing protocol.

Likewise, this demonstrates that ferritin test results are not missing at random and thus methods to impute ferritin results in the context of predictive model development are likely to be biased.

**Figure S1: Association Between Probability of Getting A Ferritin And The Ferritin Result**

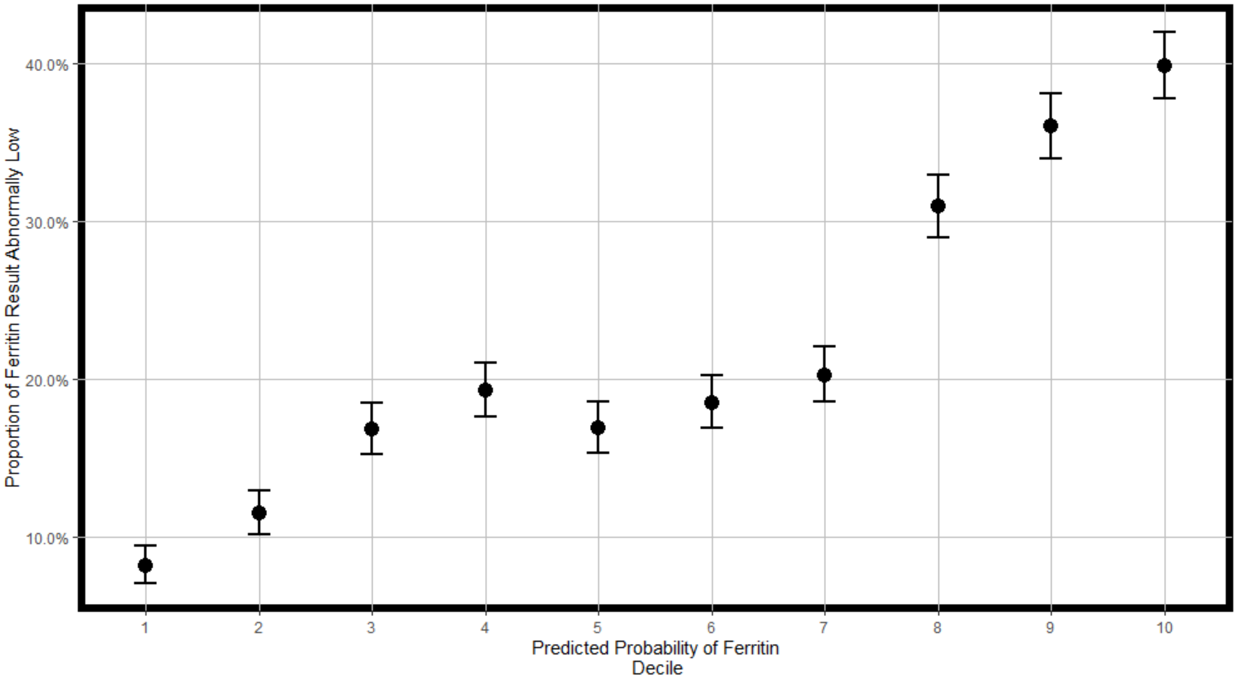


Shown is the relationship between predicted probability of ferritin (x-axis) and the probability that ferritin will be abnormally low (y-axis) for cases where ferritin was actually ordered. Error bars represent 95% confidence intervals. Spearman rho assessing the correlation between predicted probability of ferritin (by decile) and proportion of abnormal results was 0.96.

**Supplement B: Refinement of Ground Truth Labels**

After completing our primary work, we discovered that in 541 cases (~0.2%) the ground truth ferritin-ordered labels, while literally correct per the criteria defined in the methods section, were arguably misleading. In particular, these cases were labeled as not having a ferritin within 30 days after the CBC, but the patient indeed had a ferritin shortly *before* (usually 1-3 minutes) the CBC, presumably artifactually related to tube label print times. Given the small number of cases affected, we expected the impact to be minimum. Nonetheless out of an abundance of caution, we re-ran the analysis labeling cases where the ferritin was collected up to 1 hour prior to the CBC as having a ferritin test concurrent

or within 30 days. As expected, results were not materially changed (updated auROC = 0.729±0.004, auPRC= 0.349±0.01, Brier Loss: 0.095±0.003).
